# A Rare Presentation of Common Arterial Trunk with Intact Ventricular Septum

**DOI:** 10.3390/jcdd7040043

**Published:** 2020-10-12

**Authors:** Diane E. Spicer, Thora S. Steffensen

**Affiliations:** 1 Department of Pathology, Tampa General Hospital, Tampa, FL 33594, USA; Thora.Steffensen@gmail.com; 2 Division of Pediatric Cardiology, University of Florida, Gainesville, FL 32611, USA

**Keywords:** common arterial trunk, intact ventricular septum, ventriculo-arterial septal defect, outflow tract development

## Abstract

Common arterial trunk is a rare anomaly on its own, but with an intact ventricular septum it is extremely rare. An unexpected finding at autopsy prompted a review of the literature and a review of the developmental considerations associated with the outflow tracts. The case presented was an intrauterine fetal death at 37 weeks gestation. At autopsy, the only anatomic abnormalities were pulmonary dominant common arterial trunk with an intact ventricular septum, ventriculo-arterial septal defect, coarctation and widely patent arterial duct. A review of the literature and the developmental concepts related to the outflow tracts of the developing heart demonstrate the rare nature of this particular variation of common arterial trunk.

## 1. Introduction

Utilizing pure descriptive terms proves to be the most sufficient method in defining the anatomy of a common arterial trunk. The initial classification put forth by Collett and Edwards [1], and subsequently modified by Van Praagh and Van Praagh [2], leaves gray zones within the alpha-numeric classifications, although Van Praaph and Van Praagh did recognize that a common arterial trunk could co-exist with an intact ventricular septum. Several anatomic variations have subsequently been presented in the literature. The cases presented by Carr et al. [3], McElhinney et al. [4], Garg et al. [5] and Ajami et al. [6] have anatomy that is similar to the case being presented. The case presented by Carr and colleagues appears to have a very small ventricular septal defect with the dysplastic valvar leaflets overriding the edges of the defect, but not forming dual orifices. The common arterial trunk in each of the remaining cases had an intact ventricular septum with a plane of tissue arising from the crest of the muscular ventricular septum and separating the pulmonary and aortic components of the valve. The anatomy of the valves showed some variation, with the majority having a dysplastic appearance. All of the cases had right and left ventricles of relatively equal size with marked hypertrophy. Zeevi et al. [7], in contrast, presented a case with a hypoplastic right ventricle, intact ventricular septum, and a common arterial trunk arising exclusively from the left ventricle. This case also demonstrated a patent, stenotic right atrioventricular connection, and coronary arterial fistulous connections with the right ventricle. The case presented by Alves and Ferrari [8] showed a common arterial trunk arising exclusively from the right ventricle, with a hypoplastic left ventricle and an absent left atrioventricular connection. An interrupted aortic arch, atrial septal defect, and tricuspid valvar dysplasia were also described. A most interesting arrangement is described by Tsang et al. [9]. Their patient had a ventriculo-arterial septal defect, with separate aortic and pulmonary valves, but with a common ventriculo-arterial junction. 

The cases listed above represent rare variations of a congenital cardiac malformation, namely common arterial trunk, that is a rare malformation in and of itself. This sets the stage for a discussion of the developmental considerations associated with the outflow tracts, often considered ‘cono-truncal’ malformations, and the differences between common arterial trunk, aortopulmonary window, and the juxtaarterial ventricular septal defect. Our case is differentiated from aortopulmonary window because there is a common intrapericardial arterial trunk, along with a common ventriculo-arterial junction. It does not show two separate arterial valves guarding separate ventriculo-arterial junctions. Although developmentally similar to those hearts with juxtaarterial ventricular septal defects, where there is hypoplasia of the proximal outflow cushions, our case demonstrates hypoplasia of the proximal outflow tract, but with an intact ventricular septum. Probably the most interesting observation is the presence of a pulmonary component and an aortic component on either side of the fibrous partition that is in direct communication with the crest of the ventricular septum. The anatomic characteristics of the aortic and pulmonary components, however, have a very different appearance, and are somewhat different from what has been previously described. This feature also distinguishes our case, in a small way, from those described earlier. The essentially common valves in the previously described cases all had three or four leaflets, with variation in the leaflets bridging the fibrous partition to form a valvar structure shared between the right and left ventricular outlets. In our case the subpulmonary infundibulum was represented, and although hypoplastic, it demonstrates the developmental independence of the proximal component from the intermediate and distal components of the outflow tract. The intermediate component demonstrates its own independence with separate aortic and pulmonary valvar components arising on either side of the fibrous partition at the common ventriculo-arterial junction. This leaves the door open to look even deeper into the way in which the outflow tracts develop, and presents yet another variation in this group of exceedingly rare lesions. Comparisons between a common ventriculo-arterial junction and a common atrioventricular junction will be drawn.

## 2. Case Report

**Figure 1:**
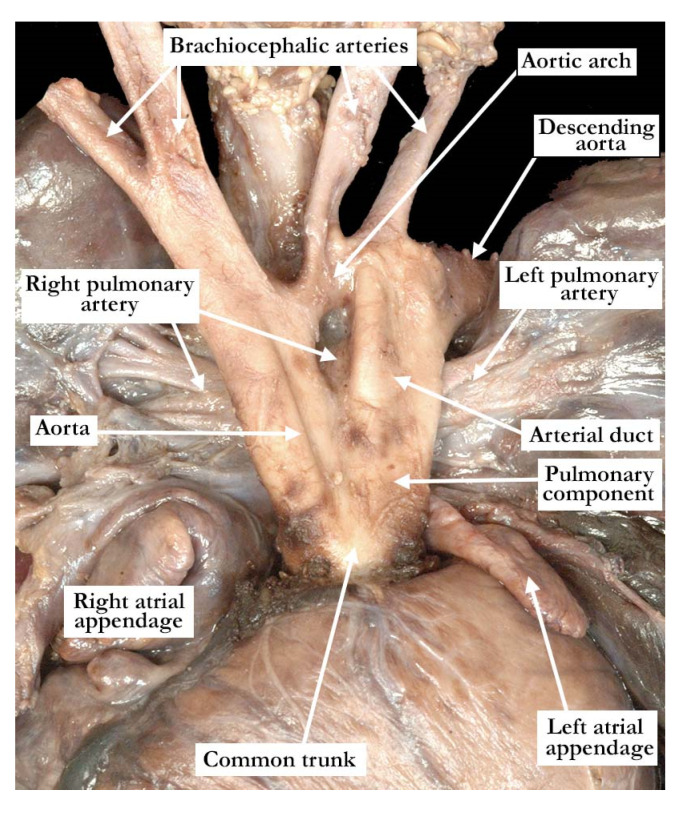
This anterior view demonstrates the common arterial trunk arising from the ventricular mass. The right and left pulmonary arteries arise separately from the posterior lateral aspects of the trunk, and there is a widely patent arterial duct. The aortic arch is hypoplastic, with the left subclavian artery arising in juxtaductal position.

The intrauterine fetal death occurred at 37 weeks of gestation, with the fetus born to a 31 year old mother who had received adequate prenatal care. The finding of a common arterial trunk, particularly with an intact ventricular septum, was an unexpected finding at autopsy. Petechial hemorrhages were noted over the lungs and the anterior surface of the right ventricle, with hemorrhage in the fat within the atrioventricular grooves. Initial examination revealed an enlarged heart with a common arterial trunk arising from the base of the heart. (Figure 1) 

**Figure 2:**
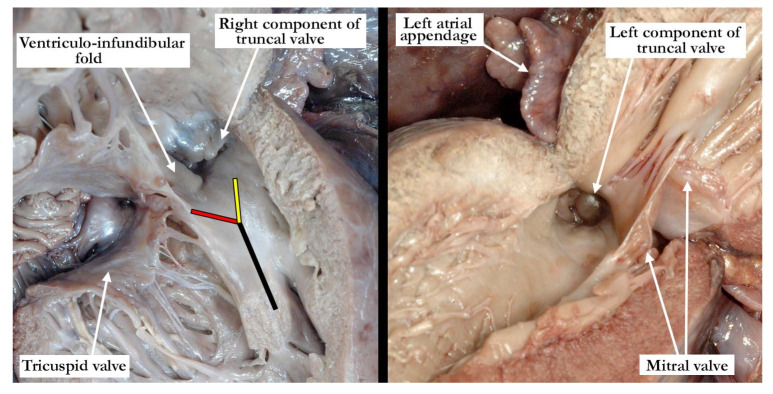
The left panel shows the right ventricle opened in clam-shell fashion with the tricuspid valve guarding the inlet and the stenotic valve at the outlet. Note the somewhat broad septal band (black line) that gives rise to the caudal (red line) and cephalic (yellow line) arms. The ventriculo-infundibular fold is hypoplastic, and inserts into the ‘Y’ of the septal band. The right panel shows the left ventricular outflow tract with the stenotic, dysplastic valve guarding the outlet. Note the hypertrophy within both the right and left ventricles.

The common arterial trunk was pulmonary dominant, with the right and left pulmonary arteries branching separately from its posterior lateral aspect. The aortic arch was hypoplastic, with the hypoplastic segment extending from the brachiocephalic trunk to the isthmus at its junction with a widely patent arterial duct. The brachiocephalic arteries had a normal branching pattern, with the left subclavian artery arising in juxtaductal position. The aortic arch extended leftward. The left brachiocephalic vein was normal, and the superior and inferior caval veins connected with the right atrium in the usual fashion. The pulmonary venous drainage was normal. The oval foramen was probe patent, and the coronary sinus was of normal caliber. Concordant atrioventricular connections were observed, with hypertrophy of the atrial and ventricular chambers secondary to the severe stenosis at the ventricular outlets. The heart weighed 37.6 g, with the normal expected weight being 16 g. The myocardium throughout both ventricles was pale. The interventricular septum was intact, and there was a common ventriculo-arterial junction. The truncal valve was divided into right and left orifices by a partition of fibrous tissue that arose from the crest of the ventricular septum and separated the pulmonary and aortic components. The partition of fibrous tissue was up to 0.4 cm in length from the crest of the muscular ventricular septum to the valvar components. The pulmonary component (Figure 2, left panel) was committed to the right ventricle, and the aortic component (Figure 2, right panel) to the left ventricle. 

**Figure 3:**
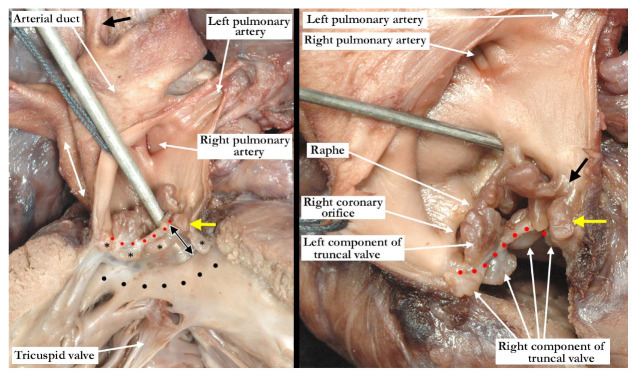
In the left panel, the anterior view of the common trunk is viewed through the opened, right component of the valve, demonstrating the separate right and left pulmonary arteries and the ascending aortic component of the trunk (double headed white arrow). The patent arterial duct is large, with the left subclavian arising in juxtaductal position (black arrow). The probe is within the left ventricular outflow tract and can be seen through the fibrous partition that lies between the valvar components and the crest of the ventricular septum (double headed black arrow). The red dots mark the upper margin of the fibrous partition. The subvalvar infundibulum (black dots) within the right ventricle is hypoplastic. The nodular valvar tissue surrounding the right ventricular outlet to the right side of the fibrous partition is marked with black asterisks. The right panel is an anterior superior view of the truncal valve with the fibrous partition marked with red dots. The nodular right component lies inferior to the dysplastic left component. The right coronary arterial orifice lies midway between the attachment of the anterior-most leaflet to the fibrous partition and the incomplete interleaflet triangle or raphe. The probe is within the left coronary arterial orifice, which lies adjacent to the well-formed interleaflet triangle between the posterior most and leftward leaflets. The black arrow marks the area reminiscent of a broad commissure, this area directly above the fibrous partition. The yellow arrow marks the dysplastic nodule that overrides into the right ventricular component. Note the elliptical nodule on the left side of the partition. Both components of the valve are stenotic, most marked on the left.

The pulmonary component of the valve was stenotic and composed of multiple dysplastic nodules of valvar tissue dispersed in roughly annular fashion at the ventriculo-arterial junction. This arrangement is reminiscent of the valvar tissue typical for those cases that have so-called “absent pulmonary valve”. The aortic component of the valve was markedly stenotic and dysplastic. It was bicuspid, one of the sinuses being shallow and elongated, with an evident raphe or incomplete interleaflet triangle at its base. The raphe was between the right anterior leaflet and the posterior-most leaflet. The other sinus was short and quite deep. The right coronary orifice arose from the right anterior-most leaflet, and was unobstructed. This right anterior-most leaflet was adjacent to the fibrous tissue that divided the aortic and pulmonary valvar components, the leaflet partially riding on the fibrous partition (Figure 3, left panel). The leaflet associated with the short, deep, leftward sinus did not override the fibrous partition, with one well-formed interleaflet triangle between it and the posterior-most leaflet, along with an apparent commissure immediately adjacent to the fibrous partition. There were two nodular structures, very similar to the remainder of the aortic component of the valve, that lay in left anterior lateral position. One of these overrode the fibrous partition, while the other impinged on the pulmonary component. There was no valvar sinus associated with these nodular structures, although distal to the fibrous partition there was an area reminiscent of a broad commissure. The left coronary arterial orifice arose immediately adjacent to the commissure between the posterior-most and leftward sinuses. It is noteworthy that both coronary orifices arose from the aortic component of the common truncal valve. On the left side of the fibrous partition, there was an adherent and single dysplastic nodule of valvar tissue that had no associated valvar sinus (Figure 3, right panel).

In the normal situation the arterial valves are supported within separate arterial roots. It is important to understand that the right and left halves of the valve in the reported case are supported within a common arterial root. Although separated into right and left components, however, they cannot be considered to be ‘normal’. The fibrous partition represents hypoplasia of the proximal outflow cushions and effectively divides the right and left ventricular outflow tracts. The ventricular septum is intact, with no shunting possible at the ventricular level, but is not normal. This particular arrangement at the ventriculo-arterial junction can be directly compared to defects at the atrioventricular junction. A subsequent discussion will be provided.

The study was conducted in accordance with the declaration of Helsinki. An autopsy permit was appropriately signed.

## 3. Discussion

During embryonic development of all structures throughout the body, there are certainly many small deficiencies or abnormal pathways occurring to give us what we refer to as ‘variation of normal’, but do not significantly affect the function of the organ or organ system. Recent study of the ‘normal’ heart is revealing many anatomic variations that are not considered, at this point in time, as being abnormal or causing abnormal function. This is especially important with regard to the cardiac valves, with minor variations potentially affecting assessment of the mortality and morbidity of certain interventional procedures. That being said, the cases of common arterial trunk in the setting of an intact ventricular septum represent the opposite end of the developmental spectrum, where development takes an extreme deviation from normal.

Developmentally, as in the postnatal heart, it is optimal to describe the outflow tracts in a tripartite fashion, noting the proximal, intermediate and distal segments, all of which are intrapericardial. In the past, the development of the outflow tracts was described with relationship to the ‘truncus’ and the ‘conus’, but with little agreement on what represents these entities [10]. Thus, within the current ‘conotruncal’ classification, the development of the arterial valves is ignored. The valves develop within the intermediate segment, or the middle, of the developing outflow tract. This obvious fact raises the question as to why abnormalities of the arterial valves are not considered to be ‘conotruncal’ malformations? Developmentally, the arterial roots, made up of the valvar leaflets and their supporting sinuses, make up the intermediate segment of the outflow tract. It is the proximal segment that forms the ventricular outflow tracts, while the distal segment becomes the intrapericardial arterial trunks.

In the recent past, the study of mouse embryos using episcopic datasets has provided new insight on cardiac development, including the arterial valves [11,12,13,14]. It has been shown that ingrowth of nonmyocardial components, coupled with migration of neural crest cells, plays an important part in the formation of the intrapericardial arterial trunks and the arterial valves. The distal separation of the aorta and pulmonary trunk commences with growth of nonmyocardial tissue into the pericardial cavity, which will form the parietal walls of the two arterial trunks. The aortopulmonary septum, when first seen, originates as a ventral protrusion from the dorsal wall of the aortic sac. It will grow in a caudal direction, extending toward the cushions that have developed at the same time within the component of the outflow tract that retains its myocardial walls. The protrusion will eventually fuse with the distal outflow cushions, thus obliterating the embryonic aortopulmonary foramen. With obliteration of the foramen, the intrapericardial outflow tract becomes separated into the aorta and pulmonary trunk. Closure of the embryonic aortopulmonary foramen is also dependent on the appropriate migration of cells derived from the neural crest. It is abnormal formation of the aortopulmonary septum that will underscore the known variations of aortopulmonary window, along with the intrapericardial pathways found in the setting of common arterial trunk. The intrapericardial pathways refer to the portion of the developing outflow tract that is distal to the sinutubular junction, but which remains within the confines of the pericardial cavity.

During the process of obliteration of the aortopulmonary foramen, however, the distal cushions have themselves fused in what is now the intermediate portion of the outflow tract, along with appearance of the intercalated valvar swellings. The intercalated swellings are located parietally within the intermediate part of the outflow tract, which retains its myocardial walls. They are offset, with one located more cranially than the other. Cavitation commences at the distal margin of the fused major cushions, along with the intercalated swellings, thus forming the primordiums of the aortic and pulmonary valves. If the major outflow cushions within the intermediate portion of the outflow tract fail to fuse, this results in a common arterial valve. The variation in the number of leaflets seen in those hearts with common arterial trunk are dependent either on the failure of fusion, or hypoplasia, of the cushions and swellings found within the intermediate portion. As emphasized, the myocardial walls of the intermediate portion, at this point, are retained. As cavitation continues, nonetheless, there is continuing ingrowth of nonmyocardial tissue from the second heart field. The cushions and swellings will remodel as cavitation continues, becoming the valvar leaflets. The added non-myocardial tissue from the second heart field then forms the sinuses of the arterial roots. As development continues, the central component of the fused cushions, initially derived from neural crest cells, undergoes apoptosis to result in separation of the aortic from the pulmonary root. At this point, the shell of the cushion mass is also undergoing myocardialisation. 

As development progresses, the proximal outflow cushions will also fuse with one another, and with the crest of the ventricular septum. This process creates a shelf within the roof of the right ventricle, placing the aortic root in continuity with the cavity of the left ventricle. The shelf will eventually become the subpulmonary infundibulum, and will lift the pulmonary valve above the aortic root. The central core of these cushions is also populated with neural crest cells that will undergo apoptosis, thus creating the extracavitary space between the muscularized subpulmonary infundibulum and the sinuses of the aortic root. Maldevelopment or hypoplasia of the proximal outflow cushions results, most commonly, in the retention of the juxtaarterial ventricular septal defect. The proximal cushions, of course, themselves usually fail to fuse in the setting of common arterial trunk. 

Indeed, the fact that failure of fusion of the outflow cushions was the reason for persistence of the common arterial trunk was demonstrated in an animal model, as long ago as 1978 by Van Mierop and his colleagues studying Keeshond dogs [15]. Most recently, the study of episcopic data sets in the mouse have shown similar models to clarify many of the developmental pathways. The knowledge of embryologic development of many different congenital cardiac malformations has significantly increased with the availability of the 3-dimensional models, but they fail to replicate many of the variants that are seen in the clinical setting. With advances in molecular biology and genetics, however, it is likely that the clinical variations can be explored with greater specificity in the animal models knowing the anatomical variations that can occur. 

Our case, however, represents a variant of common arterial trunk with an intact ventricular septum that differs from the cases previously noted in the literature. As described in all of the similar cases, there was a fibrous partition that separated the right from the left components of the outflow tracts. This fibrous partition is reminiscent of the fibrous raphe that is often appreciated between the conjoined leaflets, and is to be found in the roof of the doubly committed and juxtaarterial ventricular septal defect. The raphe, and the fibrous partition described in our case, are most likely derived from the proximal cushions which have failed to muscularise. On either side of this fibrous partition, our case showed valvar components that had a significantly different appearance from one another. The tissue to the right is very reminiscent of the valvar tissue observed in those cases described as having absent pulmonary valve. The nodular remnants of valve tissue encircled the right ventricular outflow in annular fashion and did not bridge the fibrous partition. There was, furthermore, formation of a muscular infundibulum. The aortic component, on the other hand, had a very dysplastic appearance, with recognizable leaflets and valvar sinuses. As would be expected, the subvalvar infundibulum within the right ventricle is hypoplastic. It is interesting to note that the right component of the valve was actually slightly inferior to the left component (see Figure 3, right panel). This is consistent with hypoplasia of the proximal outflow cushions. 

Common arterial trunk is frequently associated with DiGeorge Syndrome (del 22q11.2), but the chromosomal microarray in our case revealed a chromosomally normal female. Our case, therefore, is an example of isolated congenital heart disease with no other associated abnormalities. As previously stated, common arterial trunk is a rare anomaly, comprising no more than 1% of all occurrences of congenital heart disease. Nearly half of all patients with common arterial trunk, however, present with some associated genetic abnormality, a syndrome, or other noncardiac abnormality. 

## 4. Conclusions

**Figure 4:**
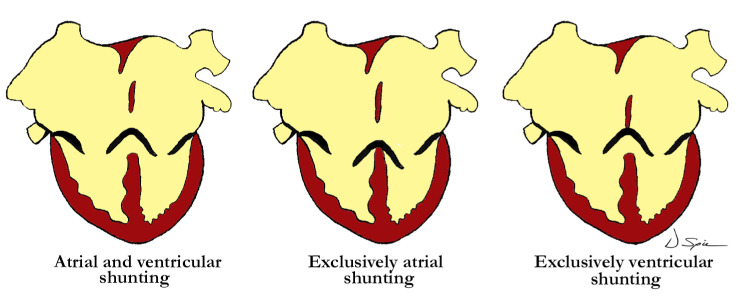
The illustration demonstrates the levels of shunting associated with the valvar arrangements in those hearts with atrioventricular septal defect. The left image shows both atrial and ventricular shunting, in the center there is exclusively atrial shunting, and to the right, exclusively ventricular shunting.

**Figure 5:**
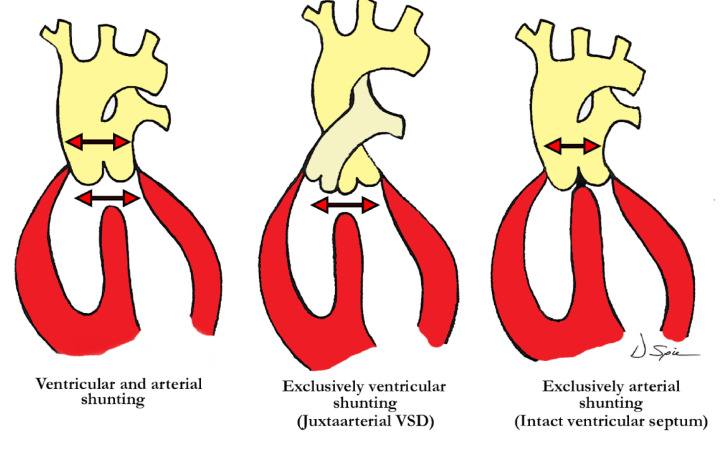
The illustration demonstrates the level of shunting in hearts with ventriculo-arterial septal defect with common ventriculo-arterial junction. The left image shows shunting at both ventricular and arterial levels, in the center there is exclusively ventricular shunting (juxtaarterial ventricular septal defect), and to the right, exclusively arterial shunting as demonstrated in our case with common arterial trunk and intact ventricular septum.

Straightforward descriptive analysis is the best path to follow when describing complex cardiac abnormalities such as the one presented here. This also applies to the developmental processes, allowing embryonic development and pre- or postnatal anatomy to be correlated in simple fashion. For those not familiar with a ventriculo-arterial septal defect, it may better be understood by comparing it to a so-called ‘ostium primum’ type of atrioventricular septal defect, where the bridging leaflets are attached to one another across the crest of the ventricular septum and effectively dividing the common atrioventricular junction into separate right and left atrioventricular orifices, as proposed in the previous description of Garg and colleagues. They draw comparison between the ventral protrusion from the dorsal wall of the aortic sac, which will form the aortopulmonary septum, and the vestibular spine, which occurs at the venous pole, and is responsible for separating the atrioventricular junction into tricuspid and mitral valves. A direct comparison can be drawn between the types of shunting that can occur with a common atrioventricular junction (Figure 4), and those hearts with a ventriculo-arterial septal defect and common ventriculo-arterial junction (Figure 5).

**Figure 6:**
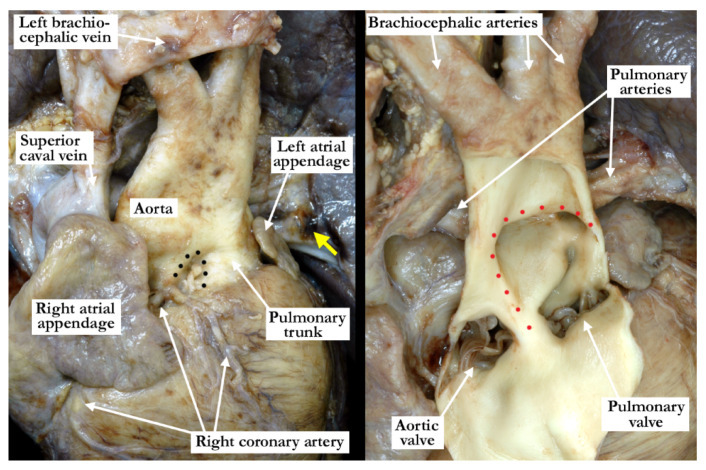
The left panel is an anterior superior view of the intrapericardial arterial trunks in a heart with an aortopulmonary window. The black dots demonstrate the separate aortic and pulmonary roots as the arterial trunks arise from the ventricular mass. (Yellow arrow—Left pulmonary artery) The right panel is from the same heart shown in the left panel. It demonstrates the large aortopulmonary window with the anterior aspect of the arterial trunks incised and folded forward. The borders of the window are marked with red dots. Note the entirely separate aortic and pulmonary valves.

The case presented shows similarity to some of those presented in the literature in that the ventriculo-arterial junction was common, there was an associated common arterial trunk, and the ventricular septum was intact. Our case has a discrete subpulmonary infundibulum, and although hypoplastic, it was indeed present. Presence of an infundibulum is proof that the proximal cushions fused and muscularized, thus the formation of separate ventricular outflow tracts, along with an intact ventricular septum. The valvar components on either side of the fibrous partition, dividing the right and left components of the outlet, are what differs most significantly between our case and the others. The other cases had three to four dysplastic leaflets shared between the outlets, and bridging, to varying degrees, over the fibrous partition. The outflow cushions within the intermediate portion of the developing outflow tract failed to fuse in all cases with incomplete remodeling of the valves and sinuses to varying degrees. Even though our case had distinctive pulmonary and aortic components, it has a common ventriculo-arterial junction which differentiates it from the two, entirely separate arterial valves associated with aortopulmonary window (Figure 6). Even though some aortopulmonary windows can be quite large, the three developmental components are typically well represented with failure of the embryonic aortopulmonary foramen to close, allowing for the defect between the intrapericardial arterial trunks. Hearts that have an aortopulmonary window most often have an intact ventricular septum.

**Figure 7:**
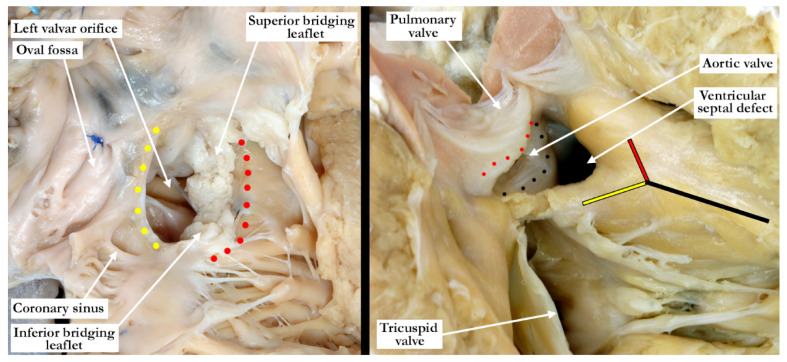
The left panel shows the opened right atrioventricular junction in this heart with a common atrioventricular junction. The superior and inferior bridging leaflets are adherent to one another and to the crest of the ventricular septum (red dots) effectively dividing the common junction into separate right and left orifices. Shunting can only take place at the atrial level. The yellow dots mark the leading edge of the atrial septum. The right panel shows the right ventricular outflow with a doubly committed, subarterial ventricular septal defect. The black line marks the body of the septal band, the red line, the cephalic arm and the yellow line the caudal arm which maintains its connection with the inner heart curvature, and thus will protect the conduction tissue. The pulmonary and aortic (black dots) valves are separate structures, and are in fibrous continuity (red dots) at the roof of the defect. Shunting can only occur at the ventricular level.

One of the typical anatomic findings occurring with a common arterial trunk is a subarterial ventricular septal defect allowing for shunting at both the ventricular and arterial levels. This is directly comparable to those cases with a common atrioventricular junction and a common atrioventricular valve where shunting is permitted at the atrial and ventricular levels. A common ventriculo-arterial junction with an intact septum can also be directly compared to a common atrioventricular junction with shunting permitted only at the atrial level, as in the so-called ‘ostium primum’ defect (Figure 7, left panel). In this situation the atrial vestibules can be discrete just as our outflow tracts are discrete. The superior and inferior bridging leaflets are adherent to one another across the crest of the ventricular septum and analogous to what we see in those hearts with common ventriculo-arterial junction and intact ventricular septum. Not only did our case have a subpulmonary infundibulum marking the presence of separate or discrete ventricular outflow tracts, the common valve also had different characteristics on either aspect of the fibrous partition that divided the common ventriculo-arterial junction. Those hearts with a doubly committed and juxtaarterial ventricular septal defect allow shunting only at the ventricular level with developmental hypoplasia of the proximal most component of the outflow tract. The intermediate and distal components are well developed in this situation with two separate arterial valves, that are in fibrous continuity with one another, and two separate intrapericardial arterial trunks (Figure 7, right panel).

The case presented shows how all three components of the outflow tract develop with great independence. Abnormalities, inhibiting the migration of the extracardiac components, which include those from the second heart field along with the neural crest cells can significantly affect the anatomy of each of the three components of the developing outflow tracts. Our case demonstrates significant differentiation at the common ventriculo-arterial junction that has not yet been reported.
